# Automated telephone follow-up after breast cancer: an acceptability and feasibility pilot study

**DOI:** 10.1038/sj.bjc.6604567

**Published:** 2008-08-26

**Authors:** D A Montgomery, K Krupa, C Wilson, T G Cooke

**Affiliations:** 1 University Department of Surgery, Level 2, Queen Elizabeth Building, Glasgow Royal Infirmary, Glasgow G31 2ER, UK; 2 Department of Surgery; Royal Alexandra Hospital, Paisley, UK; 3 University Department of Surgery, Western Infirmary, Glasgow, UK; 4 University Department of Surgery, Glasgow Royal Infirmary, Glasgow, UK

**Keywords:** breast cancer, follow-up, telephone, automated

## Abstract

Traditional clinical follow-up after breast cancer is inefficient at detecting relapse and is poorly suited to detecting and ameliorating psychological problems. There is interest in developing more effective and efficient methods of follow-up. We report a prospective cohort study of the acceptability and feasibility of remote, automated telephone follow-up after breast cancer. Women with a history of breast cancer were approached at their annual follow-up visit. For participants, the follow-up questionnaire was administered on paper at baseline. In place of a clinic visit following year, the women completed the same questionnaire using an automated telephone system. All patients were given mammograms. A semi-structured interview was then conducted to assess the acceptability. The potential impact on clinic usage was assessed. In all, 110 of 121 women (91%) agreed to participate. Seventy-five patients (71%) completed follow-up using the new automated system 1 year later. Seventy-one of the 75 patients found the system easy to use. Forty-nine of the 75 (65.33%) liked the system and were happy to use it as their sole method of follow-up. A further 12% were happy to use it as part of their follow-up. In only 10.66% of participants were concerns raised which led to clinic attendance. Automated questionnaire-based telephone follow-up is acceptable to women and has the potential to reduce attendance at clinic. Further studies to validate this method further are planned.

The National Institute for Clinical Excellence (NICE) recommend only 2–3 years of follow-up after treatment for breast cancer before discharge to the general practitioner (GP) (National Institute for Clinical Excellence, 2002). There is reluctance on the part of hospital clinicians to implement these guidelines (Donnelly *et al*, 2007). Although breast surgeons and oncologists recognise the benefit of reduced clinic workload which comes with discharging patients to general practice, anxiety over perceived lack of GP training and experience in oncology and potential loss of patient outcome data leave them reluctant to follow NICE guidance (Donnelly *et al*, 2007).

National Institute for Clinical Excellence considers that routine follow-up should be provided for the detection of locoregional relapse, provision of psychosocial support and the detection and amelioration of side effects of therapy (National Institute for Clinical Excellence, 2002). Routine clinical examination is an inefficient way of detecting potentially treatable relapse after breast cancer (de Bock *et al*, 2004). Local control after breast-conserving surgery and modern radiotherapy are excellent; event rates are low. Locoregional relapse, including new contralateral cancers, occurs with a rate of 1–1.5% of patients at risk per year in a contemporary cohort, with only 13% of such relapses detected by routine clinical examination in asymptomatic patients (Montgomery *et al*, 2007b). It is possible that this first NICE aim, of detecting locoregional relapse, may be adequately achieved by regular mammography and patient education on self-examination in our patient group (Montgomery *et al*, 2007b), although this will be confirmed in future trials.

Routine clinical follow-up appears to have little impact in terms of the additional aims as outlined by NICE. A recent review confirmed that the few randomised trials of alternative methods of follow-up in breast cancer that have been conducted have revealed no difference in quality of life in patients followed up in the traditional outpatient setting compared with any alternative (Montgomery *et al*, 2007a).

Follow-up by telephone has been tried in other disciplines. James *et al* (1994) reported good outcomes in terms of detecting medical problems and providing support to oncology patients using a nurse-led telephone system. Automated telephone follow-up has potential benefits in terms of reducing clinic visits, reducing workload for clinical staff and improving detection of psychological problems in women after breast cancer. Such follow-up could be used as a screening tool for detecting women with psychosocial problems or side effects of therapy after treatment for breast cancer, and clinic visits could then be targeted to those who require such visits. The automation could prove cost effective in reducing staff requirements. A systematic review of uses of automated telephone computer systems concluded that the reliability and availability of such systems made them attractive for use in follow-up, but that little evidence for their use in this setting existed and that further trials were needed (Biem *et al*, 2003).

It is possible that routine clinical examination is unnecessary after breast cancer and that the first aim of NICE, the detection of treatable relapse, can be achieved by regular self-examination and annual mammograms. The intention of this study is to assess whether a new automated questionnaire-based telephone follow-up system is an acceptable and feasible method for achieving the additional aims of the NICE guidelines, those of detection of side effects and psychological problems.

## Methods

### Study design

A prospective cohort study of the acceptability and feasibility of remote automated questionnaire-based telephone follow-up after breast cancer.

*Inclusion and exclusion criteria* All patients with a history of breast cancer treated with curative intent who had completed their adjuvant chemo-radiotherapy and who attended a routine follow-up clinic between 1 May 2006 and 31 August 2006 were asked to participate.

The following exclusion criteria were applied:

- The presence of local or distant disease at time of clinic. (Successfully treated previous locoregional relapse was not an exclusion criterion).
- Inability to complete the questionnaire on paper independently.
- No access to a telephone.
- Inability to consent to participation.

Inability to complete the questionnaire by phone because of physical difficulties was not an exclusion criterion if a paper version could be completed and the patient had assistance from a willing family member to complete the questionnaire over the phone.

*Design of the telephone system* An electronic case record (Excelicare™) with linked telephone system (Excelicare Direct) is available from AxSys Technology, Glasgow, UK. Questionnaire scripts can be programmed onto the system, so that patients can telephone in and complete the questionnaire using an ordinary touch tone telephone. The answers generated are recorded within an individual patient record. The system can be programmed to calculate scores according to the answers given in the questionnaire and undertake specific actions in the event of certain answers being given or scores being achieved. For example, an acceptable score can result in a reassuring letter being generated by the system and a request for a routine mammogram being sent to radiology. Poor scores or deterioration from the last-recorded score can result in an email being sent to a designated person to ensure that the low score is followed up.

The questionnaire used in this project consisted of two parts. An initial seven questions were designed to encourage self-examination and allow the reporting of symptoms that may be associated with relapse, either locoregional or metastatic. This part of the questionnaire had not previously been validated and is reproduced as Appendix 1.

The main objectives of this computer telephone follow-up system are to improve the detection of psychosocial concerns and of treatment-related side effects among our patients. The more general quality of life questionnaires that exist have been shown to be nonspecific in relation to breast cancer and often insensitive to problems in breast cancer patients (Hall *et al*, 1999). For this reason, the more breast cancer-specific Functional Assessment of Cancer Therapy-Breast (FACT-B) questionnaire with endocrine and arm subscales was used here. The characteristics of the FACT-B questionnaire has been described elsewhere (Brady *et al*, 1997; Fallowfield *et al*, 1999; Coster *et al*, 2001). Briefly, FACT-B is a 44-item self-report questionnaire designed to measure general quality of life issues as well as more specific issues related to breast cancer, and it has been extensively used in both research and clinical practice. The arm subscale consists of four additional items used in conjunction with FACT-B and has been shown to be sensitive to deterioration in arm function associated with the development of lymphoedema, as well as being sensitive to improvement in function associated with effective treatment (Coster *et al*, 2001). The endocrine subscale consists of 18 items designed to detect endocrine symptoms related to adjuvant treatment (Fallowfield *et al*, 1999). The full FACT-B questionnaire with arm and endocrine subscales is reproduced as Appendix 2.

*Protocol* Follow-up is conducted on an annual basis within our unit. At the end of a routine scheduled clinic visit, patients were invited to complete their next follow-up visit the following year using the computer telephone system.

Patients who agreed to participate completed the full questionnaire on paper before leaving the clinic to obtain a baseline score. They were informed that they would be written to 10 months later and asked to complete the same questionnaire over the telephone. The automated nature of the system was explained to them in detail. All participants were informed that they could withdraw consent and come for a standard clinic visit at any time, and were informed that this author (DAM) would telephone them after they had completed the telephone follow-up questionnaire to go over their answers with them, address any concerns they had and obtain feedback on the system.

Ten months after recruitment, all participants were written to with instructions to telephone in and complete the questionnaire. Full instructions on how to use the telephone system are included on the system and were backed up by written instructions on the letter. A paper version of the complete questionnaire was sent to all participants so that they could go over their answers beforehand if they wished.

All patients were telephoned by the author within 1 week of completing the telephone-based questionnaire. A semi-structured interview was conducted; the questions used are reproduced as Appendix 3. Any areas of low score or significant deterioration from the baseline score in any part of the questionnaire were discussed. Previous studies have suggested that the minimally important change in score in any section of the FACT-B questionnaire, which is associated with a true deterioration in symptoms important to the patient, is 5% of the total available score for that section (Cella *et al*, 2002; Eton *et al*, 2004). This was taken to be the case for this initial study. If the patient answered yes to any of the initial seven questions, this was also discussed. Problems that could not be resolved over the telephone resulted in an appointment being sent for the patient to come to the clinic. Patients were also brought back to clinic if they requested to come for clinical examination. Finally, all patients were sent appointments to attend for mammography.

## Results

In all, 110 of 121 patients (91%) invited to participate agreed and completed a baseline questionnaire. The mean age of participants was 62 years (s.d. 11.8 years), with those refusing to participate having a mean age of 68 years (s.d. 7.4 years), (*P*=NS). The characteristics of the included patients are shown in Table 1. One patient, not included in the table, had a single scalp lesion resected, which was pathologically an oestrogen receptor-positive breast cancer. Following treatment, she has now been disease-free for 5 years in routine follow-up and was keen to give feedback on the new telephone system.

Nine months after enrolment, all 110 patients who agreed to participate were cross-referenced with our recurrence database. Five patients had died since enrolment. The remaining patients were written to with instructions to telephone in and complete a follow-up questionnaire over the telephone. Non-responders were written to once more and then finally telephoned to establish reasons for non-response and to offer a standard clinic visit.

*Willingness to participate* Of the 105 patients written to, 75 patients (71%) telephoned in and completed a follow-up questionnaire. Of the patients who did not complete the questionnaire over the telephone, three (3%) were uncontactable by letter or telephone and five (5%) did not complete the questionnaire even after telephone reminder to do so but gave no reason for non-participation. Eight (8%) could not manage to use the telephone system for technical reasons, such as feeling ‘too old’, deteriorating hearing or intercurrent illness. One patient claimed that she did not have the time, one felt that it was too detailed and one patient stated that she did not want to think about her breast cancer in such detail. Only three patients (3%) did not want to complete the questionnaire because they preferred to come to clinic for examination.

The mean age of patients who completed the follow-up questionnaire using the computer telephone system was 59 years (s.d. 11 years) compared with a mean age of 67 years (s.d. 11 years) in those who did not (*P*=0.002). In all, 66.66% of women born between 1930 and 1939 successfully completed the telephone version, with only 30% of patients born between 1920 and 1929 doing so (*χ*^2^ 1, d.f. *P*=0.046).

*Ease of use and acceptability of the telephone system* Seventy-one of the 75 patients who completed the questionnaire found the computer telephone system easy to use. Two patients were anxious about using this technology, so completed the questionnaire on paper and had a member of their family call in the results. All patients stated that having the questionnaire on paper in front of them when using the computer telephone service reduced the anxiety associated with this new technology and simplified the process.

Forty-nine of the 75 patients (65.33%) who completed the computer telephone follow-up questionnaire liked the system and were happy to use it instead of routine clinic visits as their method of follow-up. Nine patients (12%) liked many aspects of the computer telephone system and stated that they would be happy to use it as a component of their follow-up. All nine of these patients stated that they appreciated the reassurance of regular clinical examination in addition. Suggestions from this group for the use of this new system included completing the questionnaire using the phone system before they came to clinic in order that their clinic visit could be more focussed or alternating between telephone follow-up and clinic visits each year to reduce the frequency of clinic visits. One patient suggested coupling this new system with clinical examination by GP.

Patients cited several benefits of this new follow-up method. In particular, time saved by the patient and the patients' families was considered beneficial. One patient stated that she was able to complete the questionnaire with her daughter and have her daughter call it in during the evening, thus saving her daughter from taking a day off work. She additionally stated that coming to the hospital and trying to find a parking space added greatly to the stress of an already stressful visit, a concern mirrored by several others. Several patients stated that not having to take time off work themselves was of benefit to them.

A number of patients reported that they found the telephone follow-up method much less stressful than coming to clinic. They were able to complete the questions in their home environment, which they found less threatening and less likely to drag up memories of their treatment. Consequently, they felt more able to think about how they were feeling in detail, particularly as they completed the paper version of the questionnaire over a longer period of time than they usually got to see their doctor in clinic before phoning in to record their answers on to the computer system.

A number of patients stated that their communication of problems within the clinic was limited by feeling embarrassed, feeling rushed or being so anxious that they forgot to mention things that had been concerning them. As a result of alleviation of all these factors, 29 of the 49 patients who were happy to use this as their sole method of follow-up felt it gave them more opportunity to divulge problems than was the case in the clinic.

Most patients described the questionnaire as being very detailed. This allowed them not only to report all the problems that they had encountered, but several women commented that, as a result of participating in this study, they felt much more aware of the type of problems that they may encounter in the future and they were now more confident that they would notice if something went wrong.

Twelve patients (16%) stated that they would not be happy to use this type of follow-up at all. Two had medical problems ongoing at the time of completion of the telephone questionnaire and suggested that their anxieties over these problems might have affected their view of the system. Four had technical problems with the system or were nervous about the new technology, including two who completed the questionnaire on paper and got their daughter to call in. These patients felt less in control than they did when attending clinic as they had to rely on family members to convey problems. Interestingly, two patients stated that the questionnaire had too much detail and they did not want to think about their breast cancer treatment in the amount of detail required to complete this type of follow-up. The rest of this group stated that they would prefer to come to clinic for an examination.

Three patients (4%) were undecided about whether they would like to use this type of follow-up. Two of these patients were at any early stage in their follow-up and felt that they would like to keep coming to clinic for a few more years until things were a bit more stable. One did not feel that the questionnaire asked the right kind of questions, but felt that clinic was not of much benefit to her either. In fact, this patient had a very detailed conversation regarding her poor scores in a number of areas of the questionnaire and it is likely that her level of anxiety required additional input. By the end of the conversation, she did concede that perhaps longer clinic appointments were needed to discuss her problems and that it had been the new telephone system that had uncovered this fact.

Finally, one patient completed the telephone questionnaire after several attempts to contact her by post and by phone. She was uncontactable after completing the questionnaire either by post or by telephone and subsequently did not attend a clinic appointment.

*Detecting problems using computer telephone follow-up* The potential impact of introducing the new computer telephone follow-up system was assessed by establishing how many patients would require to be telephoned or brought back to clinic in light of their scores on the questionnaire. As mentioned above, a change in score of 5% or more of the total score available for each section is considered a significant change in score (Cella *et al*, 2002; Eton *et al*, 2004). Patients recording deterioration in score of 5% or more would be telephoned to discuss this. Patients with issues unable to be resolved through telephone consultation would be brought back to clinic for further assessment, investigation or treatment. Patients answering yes to any of the initial seven questions outlined in Appendix one, designed to allow the patients to highlight easily any new problem they had developed in their breast or arm pit, were also telephoned to discuss the problem that had led to them answering yes and to arrange for them to be brought back to clinic if there were problems that required further investigation. Table 2 outlines the changes in scores for each of the sections for the 75 patients who completed the follow-up questionnaire using the phone system.

In total, 66 of the patients (88%) had deterioration in score in at least one section of the questionnaire of more than 5% of the total score available for that section. Forty-three patients answered yes to at least one of the initial questions shown in Appendix 1. Forty of these had also recorded deterioration in some aspect of their questionnaire and three had not. Therefore 69 patients (92%) either answered yes to one of the initial seven questions or had deterioration in their score from baseline of more than 5% in at least one section of the questionnaire. All patients were telephoned to discuss their scores.

The majority of patients stated that they felt no different at the time of the telephone questionnaire compared to when filling out the baseline questionnaire (on paper) the year before, or felt that their change in score was related to other problems in their life. In particular, a number of patients with arthritis attributed much of their deterioration in scores to joint pain, which had increased over the course of the year. One patient had suffered deterioration in her functional and emotional scores, but had already been investigated by her GP and was seeing a psychologist. All patients with deterioration in endocrine scores were given advice on dose splitting, one was sent to her GP for venlafaxine prescription and one patient was brought back to clinic to further discuss treatment options.

Four patients complained of a new bone pain that could not be dealt with satisfactorily over the telephone and all four of these patients were brought back to clinic for further treatment or investigation. One patient had had recent further axillary surgery for cosmesis and had developed some troublesome lymphoedema. She was referred to the lymphoedema clinic. Three patients brought back to investigate new lumps in the breast or axilla (all were benign) and one patient had been back for investigation of a new lump 1 week before completing the telephone questionnaire. One patient had suffered a marked deterioration in endocrine scores and complained of vaginal bleeding, but had just been diagnosed with endometrial cancer having been referred to gynaecology by her GP.

From all 75 patients who responded, only eight (10.66%) required to be brought back to clinic for further investigation or treatment of problems raised during the telephone follow-up.

## Discussion

Remote automated computer telephone follow-up has potential to reduce the burden on outpatient clinics by limiting the number of people who need to attend, while allowing clinicians to stay in charge of the ongoing management of their patients. The system allows continued generation of not only outcome data, but far more data on quality of life and side effects of therapy than would usually be available for patients out with the setting of a clinical trial.

This is the first study that we are aware in which specific quality of life tools have been used as part of the routine follow-up process in an attempt to more objectively uncover psychosocial concerns and side effects of treatment. Quality of life tools have been used to assess the impact of novel follow-up methods previously (Grunfeld *et al*, 1996, 2006; Brown *et al*, 2002; Koinberg *et al*, 2004), but never specifically to screen for problems. Using such tools in this way should be done with care. The FACT-B questionnaire and the additional arm and endocrine subscales were not developed as diagnostic tools in their own right. Yet these questionnaires do allow a very detailed assessment of patients' functioning across a wide spectrum of areas.

There are well-defined criteria as to what is likely to constitute a significant change in score, usually considered to be a change in 5% or more of the total score available for each section (Cella *et al*, 2002; Eton *et al*, 2004), and there is extensive data pertaining to the use of this questionnaire and the range of scores normally expected, although much of these data come from clinical trials and may not reflect the range of scores seen in our population.

It is difficult from this early pilot study to assess how much time could be saved by this follow-up method. In this study, all patients were telephoned by a senior doctor after completion of the questionnaire. This might have influenced their willingness to participate and their perception of the quality of the follow-up. In future, this phone call will only happen in the event of the computer system noting a deteriorating or low score, and then will be delegated to a senior nurse. It would be important to ensure that patients still found this acceptable before making definite predictions of how much resource could be saved.

In this study, most patients required to be telephoned because of reductions in scores between baseline administration of the questionnaire on paper and the telephone version completed at 1 year, but few felt that their situation had changed significantly and only eight patients needed to come up to clinic. Work is ongoing to assess the correlation between paper and telephone administration of this questionnaire. These two methods of administration are very different. Earlier study has shown that patients are more honest when reporting symptoms and so on to an automated computer telephone system than when completing questionnaires in other ways (Kobak *et al*, 1997; Turner *et al*, 1998; Millard and Carver, 1999). Technical differences between the two methods, as well as differences in the physical setting where each questionnaire was completed, may also lead to systematic difference in score between questionnaires filled out on paper compared with those by telephone. An option in future would be to have the baseline questionnaire completed remotely also.

Functional Assessment of Cancer Therapy-Breast was not developed as a diagnostic tool and so the overall scores and changes in score for an individual patient must be interpreted with caution. As mentioned by Cella *et al* (2002), the derived change in score in an individual may reflect true change, although the degree of measurement error in individual assessment precludes the use of these scores for individual diagnosis. Rather, the scores derived for any patient can be interpreted in much the same way as any screening test, as long as one keeps in mind the consequences of false-positive and false-negative results.

This study reveals automated telephone follow-up to be both feasible and acceptable to a large proportion of women. The majority of the women approached to take part in this study were happy to do so, with a randomisation rate of 91%, although it will be important to assess again how acceptable this method of follow-up is when patients are not routinely receiving a follow-up phone call and when the system is being run by senior nurses rather than by a doctor as was the case here. There was a drop-out rate subsequently, with some 30 patients (29% of patients who agreed to take part) not completing the telephone questionnaire 10–12 months after recruitment and as such it is likely that traditional clinics will continue for some people, particularly those aged over 75.

A number of women liked the technology, but were put off by not seeing a doctor face to face. For these patients, there remains the option of partnership with general practice whereby their attendance at clinic could be avoided, but they could still have some examination in primary care. This both reduces workload at clinic and has the potential to address GP concerns that they lose touch with their patient after a diagnosis of breast cancer as their care is all delivered centrally (Anvik *et al*, 2006).

Among participants, this novel method of follow-up was acceptable, with almost 80% agreeing that there were benefits to this type of follow-up over traditional clinic visits. Most of these patients felt that the process was more detailed than was usually the case during routine clinic visits. In this initial feasibility study, there was no formal assessment of the impact of this system on quality of life. We would hope this more detailed questioning would lead to greater detection of problems, a higher level of input to attempt to resolve these problems and ultimately would translate to an improved quality of life among participants in this type of follow-up process. Importantly, this more detailed questioning places no additional burden on resource when conducted using the automated phone system as all scores are calculated and data collated by the computer.

## Summary

It is likely that this system will reduce the need for routine clinic visits, but this will be offset in part by the need for a specialist nurse to oversee the telephone system, interpret patients' scores and telephone those patients who raise concerns with their telephone follow-up. This is nonetheless a more flexible arrangement than is currently available. With this new system, women can contact their secondary care team at any time without the need for clinic space and it is likely that only a small proportion will require to come to clinic. This should provide increased capacity to help meet the demand to see new patients quickly, without compromising the care of our long-term patients, and should ensure that clinic visits are more focussed on the needs of those attending.

## Figures and Tables

**Table 1:** Study population

*Age (years)*	
Mean	62
s.d.	11.8
Range	35–87
	
*Tumour grade*	No. of patients
DCIS	2
High-grade phylloides	3
1	21
2	38
3	40
Unknown	5
	
*Node status*	No. of patients
Positive	40
Negative	62
Unknown	7
	
*Procedure*	No. of patients
Conservation surgery	63
Mastectomy	31
Mastectomy/reconstruction	11
Bilateral procedures	4
	
*Adjuvant hormonal therapy*	No. of patients
Never	37
Previously	33
Current	37
Stopped between paper and phone versions	3
	
*Time in follow-up*	Time (years)
Median	5.16
Range	1–20

Age is the age of the patient at the time of recruitment to this study, and time in follow-up is the time from original diagnosis to enrolment in this study. Tumour grade, nodal status and procedure all describe initial treatment. Ajuvant hormonal therapy describes whether the patient has never or has previously had hormonal therapy, was on hormonal therapy throughout the period of this study (current) or stopped hormonal therapy between enrolment and at the end of this study.

**Table 2:** Change in scores between the baseline paper version and the telephone questionnaire completed at 10 months

	**PWB**	**SWB**	**EWB**	**FWB**	**BCS**	**ESS**	**ARM**	**FACT-B**	**FACT-G**	**FACT-ES**
Improved score	21	11	20	12	18	14	18	7	9	7
Static score	42	36	26	33	20	33	36	44	42	38
Deteriorating score	11	27	29	30	35	16	19	21	22	16
Not available^a^	1	1	0	0	2	12	2	3	2	14

ARM=arm subscale (5 items); BCS=breast cancer subscale (9 items); ESS=endocrine subscale (18 items); EWB=emotional well being (6 items); FACT-B=sum of PWB, SWB, EWB, FWB, BCS; FACT-ES=sum of PWB, SWB, EWB, FWB, BCS, ESS; FACT-G=sum of PWB, SWB, EWB, FWB; FWB=functional well being (7 items); PWB=physical well being (7 items); SWB=social well being (7 items).

Scores were considered static if the telephone score was within 5% of baseline score.

a Several patients did not complete all sections of both the baseline and telephone questionnaires and so a difference between the scores was not calculable.

## Appendix 3

Questions used for the semi-structured interview after completion of the telephone follow-up at 1 year:

- Did you find the system easy to use?
- What, if anything, did you like about this type of follow-up, compared with coming up to the clinic?
- What, if anything, did you not like about this type of follow-up?
- Do you have any ideas regarding how it could be improved?
- Do you feel sufficiently reassured by this type of follow-up? (Remembering that you would get feedback on how you scored and will also get a mammogram in the next few weeks.)
- Do you feel that the telephone questionnaire gave you more, less or just about the same opportunity to tell us about things that are worrying you compared with coming to clinic?
- Would you be happy to use this as your only method of follow-up, along with the mammograms each year?
